# Supplementary material for: Content-rich biological network constructed by mining PubMed abstracts
Source: BMC Bioinformatics. 2004 Oct 8;5:147. doi: 10.1186/1471-2105-5-147 (PMC528731; doi:10.1186/1471-2105-5-147)
Supplement: Additional File 2 — The original results of the above study (non-essential files are deleted to keep the file size under the limit set by BMC bioinformatics). [file 1471-2105-5-147-S2.bz2 › chilibotAdditionalFile2/dip30/723ID9180258E18063/html/SECG_SECY.html]

 


 **SECG** and **SECY** 
  
Found 41 abstracts in PubMed, retrieved 30.  
 

 What does Google say? 
 PDF only 
| .edu only 

---

**Interactive relationship** (e.g. stimulation, inhibition, etc)

**Neutral relationship**- These results collectively suggest that a C2 TM3 P2 TM4 C3 region of  **SecY**  serves as an interface with  **SecG** .  Ref: 12809499 Biochemistry, 2003
- The E. coli enzyme is required for proteolytic degradation of some unstable proteins that include both soluble regulatory proteins such as sigma 32 heat shock sigma factor and phage lambda CII transcriptional activator, and membrane proteins including uncomplexed forms of  **SecY**  forms the translocon together with SecE and  **SecG**  and the a subunit of the F0 complex of the H ATPase.  Ref: 10077851 FEMS Microbiol Rev, 1999
- Thus, the C2 C3 region of  **SecY**  is in the proximity of the N terminal half of the  **SecG**  cytoplasmic loop.  Ref: 12809499 Biochemistry, 2003
- These  **SecY**  Cys residues also formed a disulfide bond with an engineered cytosolic cysteine at position 28 of  **SecG** .  Ref: 12809499 Biochemistry, 2003
- Whereas homologues of Sec61alpha  **SecY**  and Sec61gamma SecE exist in archaea, identification of the third component Sec61beta or  **SecG**  has remained elusive.  Ref: 11943537 Trends Biochem Sci, 2002
- In addition, mutations in  **secG**  were isolated as suppressors against a cold sensitive mutation secY104 affecting the TM4 C3 boundary of  **SecY** .  Ref: 12809499 Biochemistry, 2003
- To facilitate large scale purification of the SecYEG heterotrimer,  **SecY**  was fused at its amino terminus with a hexahistidine tag and co overexpressed with SecE and  **SecG** .  Ref: 9425040 Biochemistry, 1998

**Non-interactive relationship** (e.g. studied together, co-existance, homology, etc.)

- Protein export in Escherichia coli is mediated by translocase, a multisubunit membrane protein complex with SecA as the peripheral subunit and the  **SecY** , SecE, and  **SecG**  proteins as the integral membrane domain.  Ref: 10074070 J Bacteriol, 1999
- In the bacterial cytoplasmic membrane, the channel is formed by oligomers of a heterotrimeric membrane protein complex consisting of subunits  **SecY** , SecE and  **SecG** .  Ref: 12167867 Nature, 2002
- Escherichia coli preprotein translocase contains a membrane embedded trimeric complex of  **SecY** , SecE and  **SecG**  SecYEG and the peripheral SecA protein.  Ref: 9184221 EMBO J, 1997
- This SRP, assisted by its receptor, FtsY, mediates the transfer to a common translocation site in the inner membrane that contains SecA,  **SecG** , and  **SecY** .  Ref: 10514469 J Biol Chem, 1999
- i Formaldehyde cross linking of translocase reveals cross links between  **SecY** , SecE and  **SecG** , but not higher order oligomers.  Ref: 10944122 EMBO J, 2000
- Escherichia coli preprotein translocase comprises a membrane embedded hexameric complex of  **SecY** , SecE,  **SecG** , SecD, SecF and YajC SecYEGDFyajC and the peripheral ATPase SecA.  Ref: 9305629 EMBO J, 1997
- **SecY** , SecE and  **SecG**  form a heterotrimer, which functions as a protein translocation channel in Escherichia coli.  Ref: 12655407 Mol Genet Genomics, 2003
- To systematically determine the functional exchangeability of translocase subunits, all of the relevant combinations of the E. coli and B. subtilis  **secY** , secE, and  **secG**  genes were expressed in E. coli.  Ref: 10559168 J Bacteriol, 1999
- Preprotein translocation in Escherichia coli is mediated by the translocase with SecA as peripheral ATPase and  **SecY** , SecE, and  **SecG**  as membrane domain.  Ref: 9425040 Biochemistry, 1998
- E. coli SecA, but not B. subtilis SecA, supported efficient ATP dependent translocation of the E. coli precursor OmpA proOmpA into inner membrane vesicles containing the hybrid SecYEG complexes, if E. coli  **SecY**  and either E. coli SecE or E. coli  **SecG**  were present.  Ref: 10559168 J Bacteriol, 1999
- SecD, SecF,  **SecG** , and  **SecY**  were decreased 4 6 fold.  Ref: 9153216 J Biol Chem, 1997
- Precursor protein translocation across the Escherichia coli inner membrane is mediated by the translocase, which is composed of a heterotrimeric integral membrane protein complex with  **SecY** , SecE, and  **SecG**  as subunits and peripherally bound SecA.  Ref: 9294424 J Bacteriol, 1997
- However, despite such a dramatic reduction in  **SecY**  and  **SecG**  levels, these membranes exhibited 50 to 70% of the wild type translocation activity, including the processing of the signal peptide, of OmpA precursor proOmpA.  Ref: 9393703 J Bacteriol, 1997
- Integral membrane components  **SecY** , SecE, and  **SecG**  of protein translocase form a complex in the Escherichia coli plasma membrane.  Ref: 12809499 Biochemistry, 2003
- YidC could not be integrated in the absence of  **SecY** , SecE, or  **SecG** , suggesting that YidC, in contrast to its mitochondrial orthologue Oxa1p, cannot engage a SecYEG independent protein conducting channel.  Ref: 11777926 J Biol Chem, 2002
- These prokaryotic eukaryotic proteins include 1  **SecY**  Sec61alpha, 2 SecE Sec61gamma, 3  **SecG**  Sec61beta, 4 Ffh SRP54 and 5 FtsY SRP receptor subunit alpha.  Ref: 12507766 Biochim Biophys Acta, 2003
- Translocation occurs at a multisubunit membrane bound enzyme termed translocase, which consists of a peripheral preprotein binding site and an ATPase domain termed SecA, a core heterotrimeric integral membrane protein complex with  **SecY** , SecE and  **SecG**  as subunits, and an accessory integral membrane protein complex containing SecD and SecF.  Ref: 10066476 Curr Opin Microbiol, 1998
- Experiments using spheroplasts revealed the physical proximity of P2  **SecY**  and the C terminal periplasmic region of  **SecG** .  Ref: 12809499 Biochemistry, 2003
- SecYEG, a complex of integral membrane proteins  **SecY** , SecE, and  **SecG**  in the cytoplasmic membrane, constitutes a pathway for polypeptide movement.  Ref: 12812064 Yi Chuan Xue Bao, 2003
- Bacterial protein translocation is mediated by translocase, a multisubunit membrane protein complex that consists of a peripheral ATPase SecA and a preprotein conducting channel with  **SecY** , SecE, and  **SecG**  as subunits.  Ref: 10559168 J Bacteriol, 1999
- Membrane vesicles prepared from these cells, when grown at the nonpermissive temperature, contained about 5%  **SecY**  and similarly low levels of  **SecG** .  Ref: 9393703 J Bacteriol, 1997
- Deletion of the N terminal region of SecE also rendered the SecE  **SecY**   **SecG**  complex unstable.  Ref: 11129584 Biosci Biotechnol Biochem, 2000
- We now report that both prlA and prlG mutations weaken the associations between the  **SecY** , SecE and  **SecG**  subunits of the translocase.  Ref: 10369667 EMBO J, 1999
- Escherichia coli preprotein translocase comprises a membrane embedded trimeric complex of  **SecY** , SecE and  **SecG** .  Ref: 11867527 EMBO J, 2002
- Upon release of the SRP at the membrane, the targeted nascent proteins insert into a translocon that contains at least SecA,  **SecY**  and  **SecG** .  Ref: 9564033 EMBO J, 1998
- Experiments using inverted membrane vesicles revealed specific cross linkings between a cysteine residue placed in the C2 or C3 domain of  **SecY**  and the cytosolic lysine Lys26 near the first transmembrane segment of  **SecG** .  Ref: 12809499 Biochemistry, 2003
- Identification of a  **SecY**   **SecG**  Interface.  Ref: 12809499 Biochemistry, 2003
- **SecY** , SecE and  **SecG**  form the membrane embedded core complex of the Escherichia coli protein export apparatus.  Ref: 10732681 Mol Gen Genet, 2000
- While SecE and  **SecY**  are generally accepted as essential components of translocase, the role of  **SecG**  is more ambiguous.  Ref: 10732681 Mol Gen Genet, 2000
- **SecY**   **SecY**  and  **SecY**   **SecG**  contacts revealed by site specific crosslinking.  Ref: 12220653 FEBS Lett, 2002
